# Common mental disorder among family carers of demented older people in Brazil

**DOI:** 10.1590/1980-57642018dn12-040010

**Published:** 2018

**Authors:** Evelise Saia Rodolpho Duarte, Liciane Vaz de Arruda Silveira, Vanessa de Albuquerque Cítero, Alessandro Ferrari Jacinto

**Affiliations:** 1 MSc, Department of Internal Medicine, São Paulo State University (UNESP), Botucatu Medical School, Botucatu, SP, Brazil; 2 PhD, Institute of Biosciences, São Paulo State University (UNESP), Botucatu Medical School, Botucatu, SP, Brazil; 3 MD, PhD, Department of Psychiatry, Federal University of São Paulo (UNIFESP), Paulista Medical School, São Paulo, SP, Brazil; 4 MD,PhD, Department of Internal Medicine, São Paulo State University (UNESP), Botucatu Medical School, Botucatu, SP, Brazil.

**Keywords:** mental disorders, carers, aged, dementia, transtorno mental, cuidadores, idoso, demência

## Abstract

**Objective:**

Population aging is a global phenomenon associated with a rising prevalence of chronic degenerative diseases such as dementia. Dementia poses a challenge not only for patients but also their family caregivers who, in exercising this role, are at higher risk of mental illness. The present study investigated the prevalence of common mental disorders (CMD) in family caregivers of demented elderly seen at a geriatric outpatient clinic of a Brazilian teaching hospital.

**Methods:**

A cross-sectional study was conducted in which the following assessment instruments were applied: the Self Reporting Questionnaire, Zarit Burden Interview, Hospital Anxiety and Depression Scale and Mini-Mental State Examination (caregivers aged ≥65 years) plus a sociodemographic questionnaire.

**Results:**

The sample comprised 90 caregivers; 83 (92.2%) women, 51 (56.7%) married, 60 (66.7%) son/daughter of elder and 62 (68.6%) holding another job besides caring for the demented elder. Caregivers had a mean age of 57.3 (±11.7) years and mean education of 9.5 (±4.9) years; 62.2% of caregivers were diagnosed with common mental disorder, 50% exhibited anxiety symptoms, 52.2% depression symptoms and 66.7% reported burden. Caregivers with common mental disorder had higher scores on the anxiety, depression and burden scales (p<0.01). Logistic regression showed that caregivers with anxiety symptoms were 15 times more likely to present common mental disorder (OR: 15.0; 95% CI: 3.5-71.2) and caregivers with symptoms of depression were 8 times more likely to have CMD (OR: 8.0; 95% CI: 2.1-31.1).

**Conclusion:**

Results revealed a high prevalence of common mental disorder in the population studied.

## RESUMO

**Objetivo:**

O envelhecimento populacional é um fenômeno mundial que está associado ao aumento da prevalência de doenças crônico-degenerativas, como as demências. Demências desafiam não somente os pacientes, mas também seus familiares cuidadores que, como consequência, estão em maior risco de adoecimento mental. O estudo verificou a prevalência de transtorno mental comum em familiares cuidadores de idosos com demência acompanhados em ambulatórios de geriatria de um hospital-escola brasileiro.

**Métodos:**

Neste estudo transversal, os instrumentos de avaliação utilizados foram: Self Reporting Questionnaire, Zarit Burden Interview, Hospital Anxiety and Depression Scale e Mini-Exame do Estado Mental (cuidadores com 65 anos ou mais) e questionário sócio demográfico.

**Resultados:**

A amostra foi composta por 90 cuidadores; 83 (92,2%) era do sexo feminino, 51 (56,7%) casados, 60 (66,7%) filhos(as) do idoso e 62 (68,6%) possuíam alguma ocupação além de cuidar do idoso dementado. A média de idade foi 57,3 (±11,7) anos e a média de escolaridade foi de 9,5 (±4,9) anos; 62,2% dos cuidadores apresentaram transtorno mental comum, 50% apresentaram sintomas de ansiedade, 52,2% apresentaram sintomas de depressão e 66,7% apresentaram sobrecarga. Cuidadores com transtorno mental comum apresentaram escores maiores nos instrumentos de ansiedade, depressão e sobrecarga (p<0,01). Regressão logística mostrou que cuidadores com sintomas de ansiedade tiveram 15 vezes mais chances de apresentar TMC (OR: 15,0; IC_95%:_ 3,5-71,2) e cuidadores com sintomas de depressão tiveram 8 vezes mais chances de apresentar TMC (OR: 8,0; IC_95%_: 2,1-31,1).

**Conclusão:**

Resultados encontrados demonstram que, na população estudada, existe alta prevalência de transtorno mental comum.

Population aging is a global phenomenon.^1^ Between 2015 and 2050, the proportion of the world population aged over 60 years is set to rise from 12% to 22%, and by 2050, 80% of older people will reside in developing countries.^2^ Also in 2050, there will be an estimated 131.5 million older people with dementia worldwide.^3^

Dementia is characterized by cognitive and behavioral changes associated with functional decline and dependence for activities of daily living.^4^ The disease poses a challenge for patients, family members and carers.^5^ Growth in the elderly population is accompanied by an increase in the number of individuals with dementia requiring assistance for activities of daily living and self-care tasks. Consequently, the number of family members caring for dementia patients also rises.^5^^,^ 6

Looking after a relative with dementia is a complex multifactorial process associated with burden for family carers.^7^ This burden depends on several factors, such as the psychological or emotional health of the carer, presence of physical morbidities, social life, ethnicity and income, and not only on the dementia symptoms of the elderly patient.^8^ Burden among dementia patient carers is considered a public health problem.^9^ Thus, caring for a patient with dementia is associated with a host of negative consequences for mental health and a high rate of mood disorders can be found in these carers.^10^^,^ 11

Some carers suffer from distress, which manifests with anxiety, somatic and depressive symptoms, yet do not fulfill the formal criteria for depression and/or anxiety diagnosis according to the DSM-V (Diagnostic and Statistical Manual of Mental Disorders – Fifth Edition) and ICD-10 (International Classification of Diseases – 10th Revision) classifications, and are thus characterized as having common mental disorder (CMD).^12^

CMD has a functional disability comparable to that of well-established chronic conditions.^13^ These disorders present either alone or together with one or more physical disorders.^12^ Various factors were found to be associated with common mental disorders, such as lower socioeconomic status, psychological illnesses, poor reproductive health, gender disadvantage and physical ill-health. Furthermore, socio-economic deprivation has been directly associated with poor reproductive health, substance use disorders (especially tobacco) and chronic medical illnesses, which ultimately lead to common mental disorders.^12^^,^ 13 Usually, CMD patients fail to seek medical assistance, and when they do so, the nonspecific somatic complaints may lead to underdiagnosis and inadequate treatment.^13^

In Brazil, the prevalence of CMD in the general population ranges from 20% to 56%.^13^ Literature investigating CMD among family carers of dementia patients is scarce.

Therefore, the objective of the present study was to determine the prevalence of common mental disorder in family carers of older people diagnosed with dementia and to identify the clinical and sociodemographic factors associated with the occurrence of CMD.

## METHODS

A cross-sectional study of carers of dementia patients from the Geriatric Outpatient Clinic of the Health Center School of the Faculdade de Medicina de Botucatu – UNESP was conducted.

Between April 2015 and March 2017, all family carers of older people diagnosed with dementia identified from medical records on outpatient service days were invited to take part in the study. Ninety-six individuals were initially invited, 6 of whom refused to participate. Thus, a total of 90 dementia patient carers present in the waiting room for medical visits or nursing care of their family member were randomly selected for inclusion. The inclusion criteria were being a carer age ≥18 years and next-of-kin of the elder (undergoing treatment at the clinic).

After inviting the family member to take part in the study, they filled out and signed the free and informed consent form and completed the following instruments:

- Mini-Mental State Examination (MMSE) – global cognition, Portuguese version (Brucki et al.).^14^
- Self Reporting Questionnaire (SRQ-20): Screens for common mental disorder (non-psychotic) in primary health care of developing countries;^13^^,^ 15 contains 20 questions with binary responses (yes/no). The validity study of the SRQ-20 for use in Brazil established a cut-off score of ≥7 yielding sensitivity of 83% and specificity of 80%, recommending its use.^13^^,^ 15
- Zarit Burden Interview (ZBI): Measures burden in carers associated with care of patients with functional and behavioral disabilities.^16^^,^ 17 The validated Brazilian version^17^ (Scazufca, 2002) has 22 items with responses rated on a 5-level Likert scale (0=never, 1=rarely, 2=sometimes, 3=quite frequently, 4=nearly always) yielding a score of 0-88 points, where higher total score indicates greater burden.
- Hospital Anxiety and Depression Scale (HADS): Assesses 14 items divided into anxious and depressive symptom subscales.^18^ In the Brazilian validity study, for a cut-off score ≥8, the scale showed 93.7% sensitivity and 72.6% specificity for anxiety, and 84.6% sensitivity and 90.3% specificity for depression.^18^^,^ 19

Statistical analyses were performed using the SPSS v.22” and STATA/SE 14 Data Analysis and Statistical Software packages, adopting a significance level of 0.05.The associations between the categorized variables were determined using the Chi-Square test. The comparison of means of continuous variables was performed using the Student´s *t*-test, given that the data had a normal distribution according to the Shapiro Wilks test.

After the initial descriptive analysis of the distribution of frequencies and contingency tables of categorical variables and of the distribution of central tendency measures of continuous variables, the distribution of the sample was explored using “presence/absence of CMD” as the dependent variable based on categorization on the SRQ using the Chi-square test and Student´s *t*-test for continuous variables. The logistic regression model was then fitted. The level of statistical significance adopted was 0.05. The dependent variable was “presence/absence of CMD”, based on a p-value of <0.25 (associations) for inclusion of the variables into the regression model.

The study was approved by the Research Ethics Committee of the Botucatu Medical School - UNESP under CAAE permit no. 40558115.3.0000.5411. The study was explained to participants, all of whom filled out and signed the Free and Informed Consent Form.

## RESULTS

The overall profile of the sample and distribution of the sociodemographic and mental health variables according to the presence/absence of CMD are given in Tables 1 and 2.

**Table 1:** Distribution of sociodemographic and mental health variables according to presence/absence of common mental disorder (CMD) in dementia patient carers, with group comparisons.

Continuous variables				Mean (SD)	
Age (years)				57.3 (11.7)	
Education (years)				9.5 (4.9)	
MMSE				27.0 (1.9)	
		**CMD present** **N=56 (%)**	**CMD absent** **N=34 (%)**	**Total** **N=90 (%)**	p*
Gender	Female	51 (56.6)	32 (35.6)	83 (92.2)	0.6
	Male	5 (5.6)	2 (2.2)	7 (7.8)	
Marital status	Married	31 (34.4)	20 (22.2)	51 (56.6)	0.21
	Without companion (single, widowed)	25 (27.8)	14 (15.6)	39 (43.4)	
Economically active	Yes	38 (42.2)	24 (26.6)	62 (68.8)	0.20
	No	18 (20.1)	10 (11.1)	28 (31.2)	
Degree of kinship	Son/daughter	40 (44.4)	20 (22.2)	60 (66.6)	
	Spouse	6 (6.6)	5 (5.6)	11 (12.2)	0.74
	Other	10 (11.1)	9 (10.1)	19 (21.2)	
Anxiety (HADS)	Improbable (≤7 points)	16 (17.8)	29 (32.2)	45 (50)	
	Possible (8-11points)	14 (15.5)	5 (5.6)	19 (21.1)	<0.01
	Probable (≥12 points)	26 (28.9)	0	26 (28.9)	
Depression (HADS)	Improbable (≤7 points)	16 (17.8)	27 (30)	43 (47.8)	
	Possible (8-11 points)	23 (25.6)	7 (7.8)	30 (33.4)	<0.01
	Probable (≥12 points)	17 (18.8)	0	17 (18.8)	
Burden (ZBI)	Absence (≤21 points)	8 (11.1)	22 (22.2)	30 (33.3)	
	Moderate (22-40 points)	34 (37.8)	10 (11.1)	44 (48.9)	<0.01
	Moderate to Severe (41-60 points)	9 (10)	2 (2.2)	11 (12.2)	
	Severe (≥61 points)	5 (5.6)	0	5 (5.6)	

* Chi-square test. The prevalence of CMD among the carers assessed was 62.2% (N=56).

**Table 2:** Estimates and 95% Confidence Intervals (CI) of odds ratios (OR), obtained by the logistic regression model.

	OR	CI_95%_(OR)	
		Upper limit	Lower bound
HADS-Anxiety (possible or probable)	15.0	3.5	71.2
HADS-Depression (possible or probable)	8.0	2.1	31.1
Carer burden	7.2	1.9	27.2
Age (younger)	0.93	0.88	0.99

According to Table 1, with regard to overall profile, carers were predominantly female (92.3%), married (56.6%), had a mean age of 57 years (±12 years), and mean education of 9.5 years (±4.9 years). The degree of kinship with the demented elder was predominantly son/daughter (66.6%) while most carers were economically active (68.8%). Of the carers assessed, 50% had possible or probable anxiety, 52.2% possible or probable depression and 66.7% reported some degree of burden.

With regard to the distribution of variables according to presence/absence of CMD among carers, no statistically significant difference was found for any of the sociodemographic variables (gender, marital status, economically active and degree of kinship); a statistically significant association (p<0.05) between anxious or depressive symptoms or burden and the presence of CMD was found among the carers.

On the multivariate analysis, the dependent variable was “presence/absence of CMD”, based on a p-value of <0.25 (associations) for inclusion of the variables into the regression model.

The logistic regression (Table 2) revealed that carers with anxiety symptoms, as measured by the HADS-Anxiety scale (possible or probable), had a 15 times greater association with CMD (OR: 15.0; 95% CI: 3.5-71.2) and carers with depression symptoms, as measured by the HADS-Depression scale (possible or probable), had an 8 times greater association with CMD (OR: 8.0; 95% CI: 2.1-31.1). Carers exhibiting burden according to score on the ZBI had a 7.2 times greater association with CMD (OR: 7.2; 95% CI: 1.9-27.2). Younger carers had a 0.93 lower association with CMD (OR: 0.93; 95% CI: 0.88-0.99).

## DISCUSSION

Response: Previous reports have shown that carers are predominantly women,^20^ a phenomenon also observed in the present study, whose sample was 92.2% female. In a psychogeriatric service in São Paulo, 49 elderly people and their caregivers were evaluated. Akin to the present study, the impact of care was assessed using the Zarit Scale and caregivers were predominantly women, daughters or wives, where a high average burden was observed.^21^

In a Norwegian study, results indicated that having a partner with dementia was associated with lower levels of life satisfaction and more symptoms of anxiety and depression than those reported by elderly spouses without dementia. Having a partner with dementia residing in a nursing home was associated with a markedly lower life satisfaction.^22^ A national study demonstrated that neuropsychiatric symptoms and severity of cognitive decline were the major factors associated with burden in patients with AD, mostly mild-to-moderate.^23^

The prevalence of CMD among family carers of dementia patients was 62.2%, proving higher than the rate for the general Brazilian population which, according to previous studies ranges from 20% to 56%,^24^ and also greater than the rate for the global population of 29.2% reported by a systematic review.^25^ In Brazil, a 2014 study in a population of 604 patients of a primary health care service found a CMD rate of 31.4%,^26^ whereas another Brazilian study in 2013 of an elderly population from Campinas city reported a CMD rate of 29.7%.^27^

The only national study assessing the presence of CMD, involving 58 family carers of older people with dementia, found a prevalence of 46.55%. The presence of CMD was associated with the practice of sports, back pain and duration of daily care.^6^

The presence of burden was reported by 66.7% of carers in the present study. A study in India^28^ showed that carer burden was greater depending on the degree of cognitive impairment of the elder. In China, a study assessing burden of dementia patient carers found that 62% of carers reported burden associated with caring for the demented patient.^29^ A Brazilian study^6^ associated the presence of burden with occupation (retired carers had greater burden), sports (carers who engaged in sport reported greater burden), presence of back pain and duration of daily care (the longer the amount of time dedicated to daily care, the greater the burden).

A study showed the negative effects of the care situation on the physical and mental health of carers, citing the prevalence of psychiatric disorders, greater incidence in the use of psychotropic medications, greater number of somatic diseases, decline in overall clinical health, social isolation, personal and family stress, feelings of obligation to exercise the role, among others.^30^ The findings of two studies showed that the presence of emotional burnout was associated with a decline in quality of life, behavioral disorders and depressive symptoms.^5^^,^ 31 Controlling behavioral symptoms of older people with dementia has been reported as more stressing for carers than managing cognitive decline.^32^

Levels of anxiety and depression are higher in carers than in the general population.^33^ In the present study, a statistically significant association was found between individuals diagnosed with CMD and anxious and depressive symptoms.

Knowledge of these characteristics is vital for establishing more effective measures to support this population. An example intervention involving carers of older people is the running of support groups and courses based on psychoeducation.^34^^,^ 35 In this context, psychoeducational interventions contribute significantly toward improving the well-being of carers, providing an educational component covering the diagnosis, course and progression of dementia to improve competencies to cope with the disease.^35^

Response: The present study has several limitations, the first being its cross-sectional design. Selection bias may have occurred given the convenience sample drawn from a specialized outpatient clinic (geriatrics) as opposed to a general clinic, where certain carers might have had a greater likelihood of being selected for the study. In the sociodemographic questionnaire, no information was requested on the duration of daily care, the degree of cognitive impairment of the elder, the presence of BPSD or whether the patient lived with the carer.
